# Efficacy of different dose of dexmedetomidine combined with remifentanil in colonoscopy: a randomized controlled trial

**DOI:** 10.1186/s12871-020-01141-4

**Published:** 2020-09-05

**Authors:** Li Jia, Meng Xie, Jing Zhang, Jingyu Guo, Tong Tong, Yuying Xing

**Affiliations:** Department of Anesthesiology, Fourth Hospital of Hebei Medical University, No. 12, Jiankang Road, Shijiazhuang, 050000 Hebei China

**Keywords:** Colonoscopy, Dexmedetomidine, Piperidines, Analgesia, Conscious sedation

## Abstract

**Background:**

Dexmedetomidine has advantages during colonoscopy as it allows the patient to cooperate during the procedure. Few studies examined the dexmedetomidine-remifentanil combination. This study was to evaluate the effects of different doses of the dexmedetomidine-remifentanil combination in colonoscopy.

**Methods:**

This was a prospective trial carried out at the Fourth Hospital of Hebei Medical University between 02/2018 and 10/2018. The patients were randomized: group I (dexmedetomidine 0.2 μg·kg^− 1^), group II (dexmedetomidine 0.3 μg·kg^− 1^), and group III (dexmedetomidine 0.4 μg·kg^− 1^), all combined with remifentanil. The primary outcomes were the patient’s body movements during the procedure and adverse events.

**Results:**

Compared with at admission (T_0_), the SBP, HR, and RR at immediately after giving DEX (T_1_), at the beginning of the examination (T_2_), 5 min after the beginning of the examination (T_3_), 10 min after the beginning of the examination (T_4_), and at the end of the examination (T_5_) in the three groups were all reduced (all *P* < 0.05), but all were within the clinically normal range. SpO_2_ remained > 98% in all patients during the examination. Compared with T_0_, the BIS values of the three groups were decreased at T_1_ and T_2_ (all *P* < 0.05). There were no significant differences in BIS among the three groups (all *P* > 0.05). The minimum BIS value in group III was lower than in groups I and II (*P* < 0.05). The degree of satisfaction with the anesthesia effect was higher in groups II and III that in group I (*P* < 0.05). No hypotension occurred, seven patients had bradycardia, and four patients had nausea/vomiting.

**Conclusions:**

Dexmedetomidine 0.3 μg·kg^− 1^ combined with remifentanil was effective for colonoscopy and had few adverse reactions.

Chinese Clinical Trial Registry: ChiCTR2000029105, Registered 13 January 2020 - Retrospectively registered.

## Background

Colonoscopy can be performed for the screening of cancer, adenomas, and polyps, for the assessment of known or possible bleeding, and for the evaluation of possible causes of abdominal pain, gastrointestinal symptoms, and/or changes in bowel habits [1, 2]. The National Colorectal Cancer Roundtable aims to reach 80% screening prevalence in the eligible American population [3, 4], representing 5.1 million colonoscopies each year [5].

Beyond the discomfort and inconveniences associated with bowel preparation [6], colonoscopy is associated with discomfort and sometimes pain. At present, the commonly used methods are the intravenous injection of propofol, etomidate, ketamine, and other drugs to make the patient’s unconscious [7]. The disadvantage is that the patient cannot cooperate during the examination (e.g., for changing position), and medical staff is needed to assist in turning over the patient, if necessary. This may compress the patient’s stomach and abdomen, which may cause gastric reflux and aspiration, which may cause pneumonia, with morbidity and even mortality [8, 9].

Dexmedetomidine (DEX) is a new type of highly selective α2 receptor agonist. It has sedative, analgesic, and anxiolytic effects, and is known as a “wake-up sedative”. Compared with propofol and fentanyl, it provides sedation without the risk of respiratory depression and can provide cooperative or semi-rousable sedation [10, 11]. It has incomparable advantages during colonoscopy as it allows the patient to cooperate during the procedure [12–15]. Dexmedetomidine can be used with other drugs such as remifentanil to achieve deeper sedation, but few studies examined the dexmedetomidine-remifentanil combination for colonoscopy.

Therefore, this study aimed to evaluate the effects of dexmedetomidine combined with remifentanil at different doses for colonoscopy. The results could provide clues about the most optimal doses and improve the patient experience of colonoscopy.

## Methods

### Study design and patients

This was a prospective trial that was carried out in patients who were scheduled to undergo colonoscopy at the Fourth Hospital of Hebei Medical University between February 2018 and October 2018. All patients were inpatients. This study was approved by the Medical Ethics Committee of the Fourth Hospital of Hebei Medical University (2017MEC113) and written informed consent was obtained from all subjects participating in the trial. The trial was registered at the Chinese Clinical Trial Registry (ChiCTR2000029105, Principal investigator: Li Jia, Date of registration: 2020-01-13). This study adheres to CONSORT guidelines.

The inclusion criteria were: 1) ASA grade was I-II; 2) 18–75 years of age; 3) weight of 50–80 kg; 4) no obvious abnormalities in preoperative ECG, blood routine, electrolytes, and other tests; 5) no history of allergies to narcotic drugs; 6) no history of sedation, analgesics, or alcohol abuse; and 7) no mental illness. The exclusion criteria were: 1) emergency patients; 2) severe abnormalities in heart, lung, kidney, liver, and other functions; 3) sleep apnea syndrome or difficult airways; 4) bronchial asthma; or 5) recent respiratory infections.

### Grouping and intervention

**Fig. 1:**
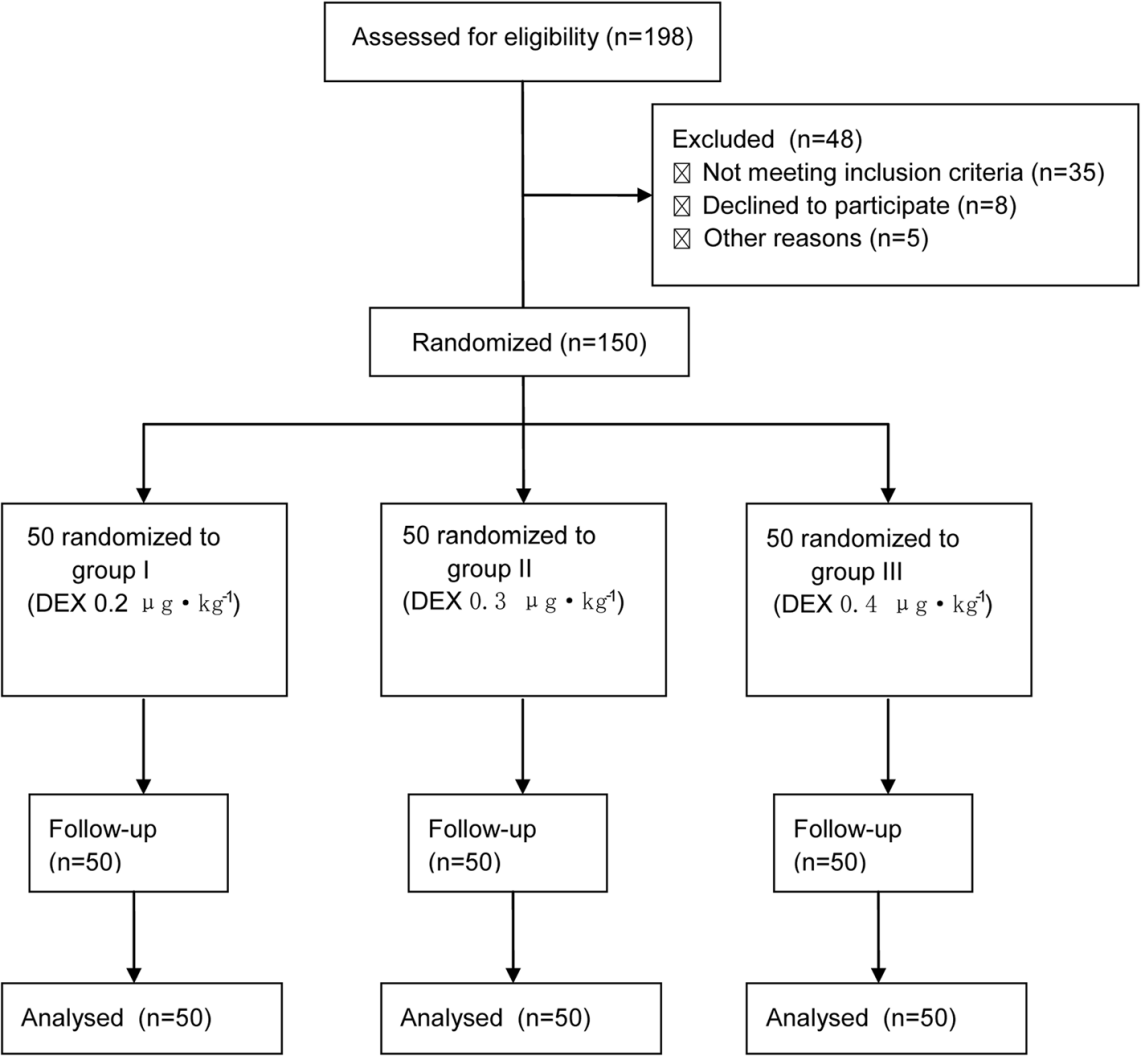
Flow Diagram

The patients were randomly divided into three groups using the random number table method: group I (DEX 0.2 μg·kg^− 1^), group II (DEX 0.3 μg·kg^− 1^), and group III (DEX 0.4 μg·kg^− 1^) (Fig. 1). Patients, surgeon and postoperative observes were blind to group allocation. The routine preoperative preparation was performed. The patient was placed on the left side with the knees bent after entering the room. The Bene View T5 monitor (Mindray Biomedical Electronics Co., Shenzhen, China) and Aspect 2000 EEG monitor (Aspect Medical Systems, Inc., Newton, MA, USA) were connected to monitor the systolic blood pressure (SBP), diastolic blood pressure (DBP), heart rate (HR), pulse oximetry (SpO_2_), respiratory rate (RR), and bispectral index (BIS). Oxygen mask inhalation was given at 5 L/min. An upper limb venous access was opened. For group I, 0.2 μg·kg^− 1^ DEX (batch number: 10122334, Jiangsu Hengrui Pharmaceutical Co., Ltd.) and a loading dose of 1 μg·kg^− 1^ remifentanil (batch number: 6120721 Yichang Renfu Pharmaceutical Co., Ltd.) was injected successively, both within 2 min with an intravenous pump. Then, remifentanil was given at a maintenance dose of 0.1 μg·kg^− 1^·min^− 1^. Colonoscopy started after 2 min. The infusion of remifentanil was stopped after the end of colonoscopy. For group II, the patients were injected intravenously with 0.3 μg·kg^− 1^ DEX. The patients in group III were injected intravenously with 0.4 μg·kg^− 1^ DEX. The dose of remifentanil was the same in all three groups. If bradycardia occurred during the examination (HR < 50 beats/min), atropine 0.5 mg was injected intravenously. Ephedrine 5–10 mg was injected intravenously if hypotension (SBP < 90 mmHg) appeared. And after the colonoscopy, patients were monitored in PACU for 30 min and transferred to the ward.

### Data collection

The data of patients, including SBP, DBP, HR, SpO_2_, and RR at admission (T_0_), immediately after giving DEX (T_1_), at the beginning of the examination (T_2_), 5 min after the beginning of the examination (T_3_), 10 min after the beginning of the examination (T_4_), and at the end of the examination (T_5_), and the BIS value at T_0_, T_1_, and T_2_, and the minimum value of BIS during the examination were recorded. We investigated whether the patient was cooperating quietly and whether there were body movements during the examination; whether the patient could wake up; if the patient was needed to turn and whether the patient could do it on his own to complete the examination; number of patients with slight limb activity who did not need additional medications; number of patients with great limb mobility who were unable to cooperate quietly and complete the examination and required additional medications (intravenous injection of 50 mg propofol); number of patients who could wake up; and number of patients who could turn over the body on their own and cooperate to the examinations were recorded. Adverse reactions such as bradycardia, hypotension, and nausea and vomiting were recorded. The examination duration was also recorded. At the end of the examination, the satisfaction degree of the surgeon on the anesthesia effect, which was divided into three grades of excellent, good, and poor, were investigated. Excellent: the patient was quiet during the examination, had no limb movement, was able to wake up during the operation, and was able to turn over the body to cooperate with the examination. Good: the patient had only slight limb movement, which did not affect the examination, was able to wake up during the operation and was able to turn over the body to cooperate with the examination. Poor: the patient had a large degree of limb activity, and it was difficult to complete the examination quietly or cooperatively, or the patient had no limb activity, but could not wake up, or could wake up but could not turn over the body to cooperate with the examination. All examinations were performed by a senior doctor in the endoscopy department of our hospital.

### Outcomes

The primary outcomes of this study were the patient’s body movements during the procedure and adverse events such as bradycardia, hypotension, nausea, and vomiting. The secondary outcomes were the duration of colonoscopy and the satisfaction of the surgeon to the anesthesia effect.

### Statistical analysis

The sample size was calculated based on the patient’s movements during the examination. Our preliminary study found that the incidence of patient movements in 11 patients with remifentanil for analgesia was 55%. A reduction of 25% after combined with dexmedetomidine was considered clinically significant. Therefore, a minimum sample size of 46 patients for each group would be required with a significance level of 5% to achieve a power of 80%. Taking into consideration a potential dropout rate of 10%, we recruited 50 patients per group. Normally distributed continuous variables were presented as mean ± standard deviation and were analysed using Student’s t test. Mann-Whitney U test was used for non-normally distributed continuous variables, which were presented as median (interquartile range) [M(Q)]. Categorical variables were expressed as frequency (percentage) and were analysed using the Pearson chi-square test. Wilcoxon rank sum test was used for comparison of rank variables. *p* < 0.05 was statistically significant.

## Results

### Characteristics of the participants

There were no significant differences in general data (age, sex, weight, and examination duration) among the three groups (all *P* > 0.05) (Table 1). There were no significant differences in SBP, DBP, HR, and RR at T_0_ among the three groups (all *P* > 0.05) (Table 2).

**Table 1:** Comparison of demographics and clinical characteristics of the three groups

Group	Cases	Age (years)	Weight (kg)	ASA (I / II)	Sex (male/female)	procedure time, min
I	50	53.4 ± 3.3	67.5 ± 2.6	20/30	27/23	20.3 ± 1.4
II	50	51.6 ± 2.5	63.3 ± 1.8	22/28	26/24	21.2 ± 1.0
III	50	55.8 ± 2.8	63.9 ± 2.6	19/31	24/26	20.7 ± 1.2

**Table 2:** Hemodynamic and respiratory changes in the three groups

Group	Items (mmHg/bpm)	T_0_	T_1_	T_2_	T_3_	T_4_	T_5_
I	SBP	125 ± 23	111 ± 22^a^	114 ± 22 ^a^	112 ± 23 ^a^	114 ± 22 ^a^	116 ± 24 ^a^
	DBP	69 ± 11	62 ± 12 ^a^	63 ± 12 ^a^	63 ± 12 ^a^	66 ± 12	69 ± 16
	HR	80 ± 14	74 ± 14 ^a^	75 ± 13 ^a^	73 ± 12 ^a^	74 ± 12 ^a^	76 ± 12 ^a^
	RR	18 ± 2	16 ± 3 ^a^	16 ± 3 ^a^	16 ± 4 ^a^	16 ± 4 ^a^	16 ± 4 ^a^
II	SBP	124 ± 20	111 ± 19 ^a^	110 ± 19 ^a^	112 ± 20 ^a^	115 ± 20 ^a^	117 ± 22 ^a^
	DBP	70 ± 11	64 ± 11 ^a^	64 ± 11 ^a^	65 ± 12 ^a^	68 ± 13	70 ± 14
	HR	73 ± 14	67 ± 12 ^a^	69 ± 15 ^a^	72 ± 16 ^a^	72 ± 16 ^a^	73 ± 16 ^a^
	RR	18 ± 2	16 ± 3 ^a^	15 ± 3 ^a^	15 ± 3 ^a^	15 ± 2 ^a^	15 ± 2 ^a^
III	SBP	123 ± 16	117 ± 21 ^a^	107 ± 18 ^a^	99 ± 18 ^a^	105 ± 19 ^a^	106 ± 18 ^a^
	DBP	72 ± 12	70 ± 14	75 ± 13 ^a^	73 ± 12 ^a^	74 ± 12 ^a^	76 ± 12 ^a^
	HR	81 ± 15	72 ± 13 ^a^	73 ± 13 ^a^	75 ± 13 ^a^	74 ± 13 ^a^	75 ± 13 ^a^
	RR	18 ± 3	16 ± 3 ^a^	15 ± 3 ^a^	15 ± 3 ^a^	15 ± 3 ^a^	15 ± 3 ^a^

^a^*P* < 0.05 vs. T_0_; all *P* > 0.05 among the three groups for all parameters at all time points. n = 50/group

### Changes in circulation and breathing parameters

**Fig. 2:**
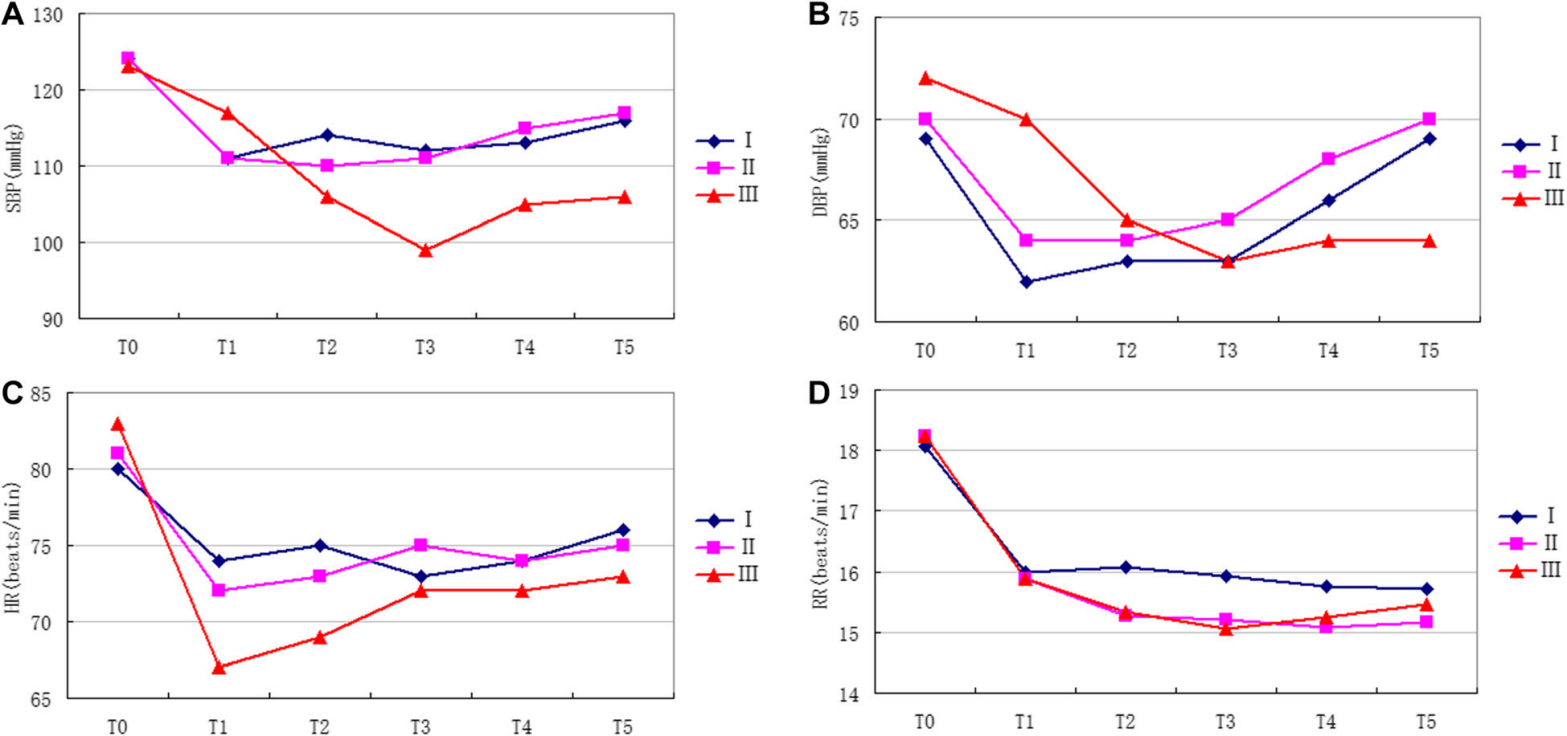
**A**: Changes of SBP of three groups of patients. **B:** Fig. 2 Changes of DBP of three groups of patients. **C:** Fig. 3 Changes of HR of three groups of patients. **D:** Fig. 4 Changes of RR of three groups of patients

Compared with T_0_, the SBP, HR, and RR at T_1–5_ in the three groups were all reduced (all *P* < 0.05), but all were within the clinically normal range (Table 2, Fig. 2). SpO_2_ remained > 98% in all patients during the examination.

### Adverse reactions

During the examination, 13, six, and three patients in groups I, II, and III, respectively, had slight body movements that did not interfere with the operation or required propofol injection (all *P* < 0.05). Two patients in group I had body movements that interfered with the operation and needed additional propofol. All patients in each group can be waked up during the examination. For patients who needed to turn over, except for one patient in group III who needed assistance, all patients could turn over on their own. There were 0, one, and six participants of bradycardia in groups I, II, and III, respectively (all *P* < 0.05). No hypotension occurred in the three groups. Nausea and vomiting occurred in one participant in group I, one in group II, and two in group III (Table 3).

**Table 3:** Comparison of the analgesic effect and occurrence of adverse events in the three groups

Items	Group I	Group II	Group III
Slight body movement	13	6^a^	3^ab^
Severe body movement	2	0	0
Could be awaked up	50	50	50
Could change position independently (patients need change position)	8 (8)	11 (11)	8 (9)
Bradycardia	0	1^a^	6^ab^
Hypotension	0	0	0
Nausea and vomiting	1	1	2

^a^*P* < 0.05 vs. group I; ^b^ *P* < 0.05 vs. group II

n = 50/group

### Bispectral index

Compared with T_0_, the BIS values of the three groups were decreased at T_1_ and T_2_ (all *P* < 0.05). There were no significant differences in BIS among the three groups (all *P* > 0.05). The minimum BIS value in group III was lower than in groups I and II (*P* < 0.05) (Table 4).

**Table 4:** Comparison of the BIS values at different time points among the three groups

Group	T_0_	T_1_	T_2_	Minimum value
I	95.1 ± 5.3	91.5 ± 8.2^a^	93.4 ± 7.2^a^	86.7 ± 7.5^a^
II	96.4 ± 2.2	92.4 ± 7.3^a^	92.5 ± 6.7^a^	84.4 ± 8.1^a^
III	97.2 ± 2.4	92.2 ± 8.5^a^	88.3 ± 9.2^a^	74.5 ± 8.3^abc^

^a^*P* < 0.05 vs. T_0_; ^b^ *P* < 0.05 vs. group I; ^c^ *P* < 0.05 vs. group II

n = 50/group

### Surgeon’s satisfaction

Thirty-five participants in group I were excellent, 13 were good, and two were poor; those numbers were 44, six, and 0, respectively, in group II; and 47, three, and 0, respectively, in group III. The degree of satisfaction with the anesthesia effect was higher in groups II and III that in group I (*P* < 0.05) (Table 5).

**Table 5:** Comparison of the satisfaction degree to the anesthesia effect of the surgeon among the three groups (*n* = 50)

Group	Excellent	Good	Poor
I	35	13	2
II	44^a^	6^a^	0^a^
III	47^a^	3^a^	0^a^

^a^*P* < 0.05 vs. group I

n = 50/group

## Discussion

Dexmedetomidine has advantages during colonoscopy as it allows the patient to cooperate during the procedure [12, 13]. Few studies examined the dexmedetomidine-remifentanil combination. Therefore, this study aimed to evaluate the effects of different doses of the dexmedetomidine-remifentanil combination in colonoscopy. The results strongly suggest that dexmedetomidine 0.3 μg·kg^− 1^ combined with remifentanil was effective for colonoscopy and had few adverse reactions.

Dexmedetomidine is a new type of highly selective α2 receptor agonist and has eight times the affinity to α2 receptors as clonidine [10, 11, 16]. Compared with clonidine, dexmedetomidine has stronger sedative, analgesic, and anxiolytic effects [10, 11, 16]. Its sedative and hypnotic characteristics are that the patients can be awakened and cooperate, and the sleep state is similar to that of natural sleep [10, 11, 16]. It is also the only sedative that allows patients to be easily awakened to cooperate without breathing depression [10, 11, 16]. Bekker et al. [17] reported for the first time the use of dexmedetomidine for craniotomy and left temporal tumor resection, during which it could be used to locate the language area and awaken intraoperatively during surgery. Ramsay et al. [18] used dexmedetomidine as the only intravenous anesthetic in the laser ablation for severe subglottic stenosis and artificial upper trachea replacement. The patients’ blood oxygen saturation was above 90% without oxygen inhalation, and hemodynamics were relatively stable. The results of this study showed that the hemodynamic parameters and respiratory parameters of the three groups of participants were within the clinically normal range, and SpO_2_ was above 98% in all participants, which was consistent with the results of the above studies.

Cortinez et al. [19] showed that dexmedetomidine had mild-to-moderate analgesic effects on cold compression tests, but had limited effects on acute pain such as electricity and thermal pain. Its analgesic mechanism is different from opioids, which can have a synergistic effect and reduce the amount of opioids [20]. Therefore, the combination of dexmedetomidine and opioids can achieve the purpose of analgesia and sedation. Remifentanil is a new type of short-acting μ opioid receptor agonist. Wilhelm et al. [21] reported that after remifentanil anesthesia, the patients could be waked up faster, and the orientation was recovered faster. Neurocognitive tests showed that it was better than fentanyl, which was more suitable for short outpatient surgery. Therefore, in this study, dexmedetomidine and remifentanil were used for painless colonoscopy. This combination has been reported before for colonoscopy [13], but the exact dose of dexmedetomidine has not been examined within the same trial.

The recommended dose of dexmedetomidine for general anesthesia is a loading dose not exceeding 1 μg·kg^− 1^. Due to the synergistic effect with remifentanil, the present study examined three doses (0.2, 0.3, and 0.4 μg·kg^− 1^) combined with remifentanil 1–2 μg·kg^− 1^ and 0.1 μg·kg^− 1^·min^− 1^ maintenance dose [22]. Remifentanil at a rate of 0.1 ± 0.05 μg·kg^− 1^·min^− 1^ did not affect ventilation and wakefulness [23]. Therefore, in this study, the loading dose of remifentanil was 1 μg·kg^− 1^, and the maintenance dose was 0.1 μg·kg^− 1^·min^− 1^. After remifentanil was given at a loading dose, the time to peak efficacy was 1.6 min [24]. Therefore, colonoscopy started 2 min after the administration of remifentanil, and dexmedetomidine also began to have effect at this time since, after dexmedetomidine infusion, the rapid distribution-related half-life is about 6 min [25].

The results of the present study showed that only two patients in group I had body movements that interfered with the examination, but the examination could be completed after the addition of propofol. Both groups II and III achieved satisfactory analgesic effects, and the patients were quiet, with no or only mild body movements, which showed that dexmedetomidine combined with remifentanil was effective for analgesia during colonoscopy. The SBP, HR, and RR of the participants during the examination in the three groups were lower than before surgery. During the examinations, the minimum BIS value in the three groups was about 73, and all patients could wake up at any time. Except for one patient in group III who needed assistance for turning over, they could turn over on their own and cooperate to change the position. At the end of the examination, the rate of excellent and good anesthesia for groups II and III reached 100%. This showed that dexmedetomidine combined with remifentanil was suitable for colonoscopy.

During the examination and after the operation, no hypotension occurred in the three groups, and the occurrence of nausea and vomiting was low, but the occurrence rate of bradycardia in group III was higher than that in groups I and II. Dexmedetomidine is a highly selective α2 receptor agonist, which activates post-synaptic α2 receptors in the central nervous system and simultaneously inhibits sympathetic nerve activity, causing lower blood pressure and heart rate.

During the examination, the minimum BIS value in groups I and II were decreased to about 86, and the minimum BIS value in group III was about 73. When all patients were waked up or talked to, the BIS value could return to more than 90. Except for one participant in group III who needed assistance, all the other patients were able to cooperate with the examiner to turn over the body on their own, and most patients did not experience any discomfort. It indicated that dexmedetomidine 0.2–0.4 μg·kg^− 1^ could produce good sedation, which was consistent with the results of Souter et al. [26]. Nevertheless, the minimum BIS value in group III was lower than those in the other two groups, suggesting that 0.4 μg·kg^− 1^ dexmedetomidine combined with remifentanil had a risk of deeper sedation when used for colonoscopy.

This trial has limitations. It was performed at a single center. There was no control group. Only three doses of dexmedetomidine were tested, without changes in the dose of remifentanil. Finally, besides BIS, no objective score was used.

## Conclusions

In conclusion, 0.3 μg·kg^− 1^ dexmedetomidine combined with remifentanil (loading dose of 1 μg·kg^− 1^, maintenance dose of 0.1 μg·kg^− 1^·min^− 1^) had a good effect, and few adverse reactions for colonoscopy.

## Data Availability

The datasets used and/or analysed during the current study are available from the corresponding author on reasonable request.

## Abbreviations

DEX: Dexmedetomidine
SBP: Systolic blood pressure
DBP: Diastolic blood pressure
HR: Heart rate
SpO2: Pulse oximetry
RR: Respiratory rate
BIS: Bispectral index
